# Can pharmacogenetics explain efficacy and safety of cisplatin pharmacotherapy?

**DOI:** 10.3389/fgene.2014.00391

**Published:** 2014-11-14

**Authors:** Ángela Roco, Juan Cayún, Stephania Contreras, Jana Stojanova, Luis Quiñones

**Affiliations:** ^1^Servicio de Salud Metropolitano OccidenteSantiago, Chile; ^2^Laboratory of Chemical Carcinogenesis and Pharmacogenetics (CQF), Molecular and Clinical Pharmacology Program, ICBM - Insituto de Ciencias Biomédicas, Faculty of Medicine, University of ChileSantiago, Chile

**Keywords:** pharmacogenetics, chemotherapy, cisplatin, polymorphisms, NER pathway, glutathione S-transferases

## Abstract

Several recent pharmacogenetic studies have investigated the variability in both outcome and toxicity in cisplatin-based therapies. These studies have focused on the genetic variability of therapeutic targets that could affect cisplatin response and toxicity in diverse type of cancer including lung, gastric, ovarian, testicular, and esophageal cancer. In this review, we seek to update the reader in this area of investigation, focusing primarily on DNA reparation enzymes and cisplatin metabolism through Glutathione S-Transferases (GSTs). Current evidence indicates a potential application of pharmacogenetics in therapeutic schemes in which cisplatin is the cornerstone of these treatments. Therefore, a collaborative effort is required to study these molecular characteristics in order to generate a genetic panel with clinical utility.

## Introduction

Cisplatin is an alkylating agent used to treat several types of cancers that works by causing DNA lesions via the formation of intrastrand and interstrand crosslinks, resulting in the activation of various signal-transduction pathways that block cellular processes, such as replication and transcription. The action of cisplatin is cell cycle-independent, although in some cases, prolonged G2 phase cell-cycle arrest occurs (Siddik, 2003; Kelland, 2007). Cisplatin has a central role in cancer chemotherapy for testicular, ovarian/cervical, head and neck, and non-small-cell cancers. The side effects include nephrotoxicity (Wong and Giandomenico, 1999), hematogenesis and neurotoxicity (Decatris et al., 2004).

**Figure 1:**
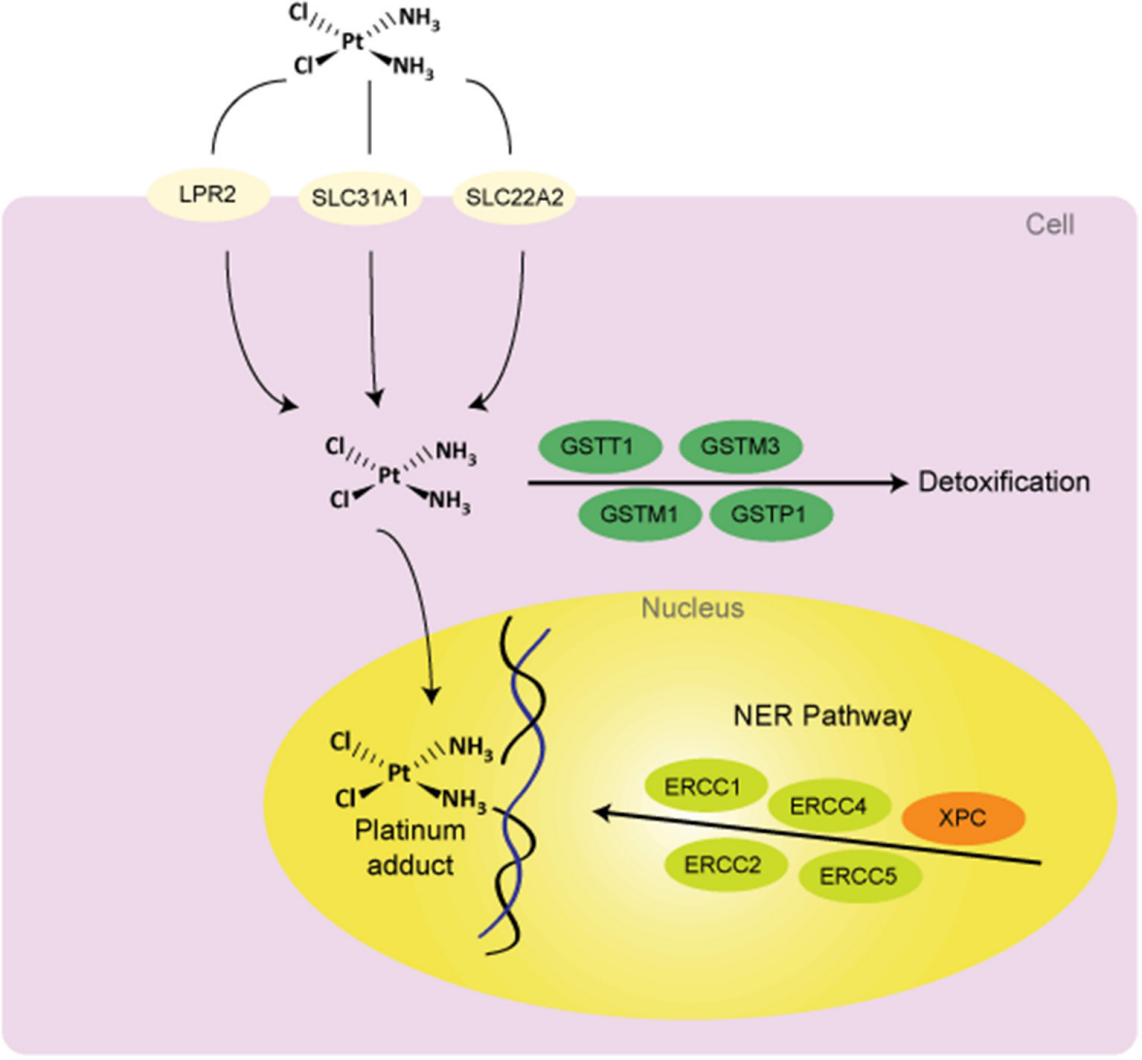
**Potential sources of variability to clinical response to cisplatin treatment**. Abbreviations: DNA, deoxyribonucleic acid; GSTs, glutathione S-Transferases; NER, nucleotide excision repair; LPR2, Low Phosphate Root2; SLC31A1 (CTR1), solute carrier family 31 (copper transporter), member 1; SLC22A2, solute carrier family 22 (organic cation transporter), member 2; ERCCs, Excision Repair Cross Complementing group of proteins; XPC, Xeroderma Pigmentosum Group C Protein.

From the beginning, cisplatin has presented variations in therapeutic response. While some tumors are hypersensitive to anticancer therapy, other tumors have an intrinsic resistance. Investigations have sought an explanation of this variation and have suggested that the major resistance mechanisms include reduction in drug levels that reach the target DNA due to reduced uptake and/or increased efflux; increased cellular thiol levels; enhanced DNA repair and/or increased damage tolerance; and failure of cell-death pathways after the formation of platinum-DNA adducts (Fojo, 2001; Siddik, 2003; Wang and Lippard, 2005). In each of these processes there exist potential sites of pharmacogenetics variability (Figure 1). Changes at the genetic level causing modifications in cellular phenotype could explain some of the variability in response and toxicity to cisplatin-included chemotherapy. In this review, we discuss associations between genetic variants in the germ line and in outcomes following cisplatin-based chemotherapy. We mainly focus on DNA repair and cisplatin detoxification through Glutathione S-Transferases (GSTs).

## Pharmacodynamic mechanisms

Cisplatin modulates several signal transduction pathways involving AKT (v-akt murine thymoma viral oncogene homolog), c-ABL (v-abl Abelson murine leukemia viral oncogene homolog 1), p53, and MAPK (mitogen-activated protein kinase)/JNK (c-Jun NH2-terminal kinase)/ERK (extracellular signal-regulated kinase). Cell death induced by cisplatin is concentration dependent and includes necrosis and apoptosis mechanisms (Gonzalez et al., 2001). Necrosis involves hyper-activation of Poly (ADP ribose) polymerase (PARP) (Nguewa et al., 2003) while apoptosis results from activation of CASP8, CASP9, CASP3, and CASP7 (Gonzalez et al., 2001).

Cisplatin distorts the structure of the DNA that generate intrastrand 1, 2—crosslinks binding proteins into shallow minor groove [high-mobility group (HMG) box proteins, repair proteins, transcription factors, histone H1] (Kartalou and Essigmann, 2001; Wozniak and Blasiak, 2002; Zdraveski et al., 2002). It covalently binds DNA and forms DNA adducts through intra- and interstrand crosslinks (ICLs). Intrastrand crosslinks are repaired by nucleotide excision repair (NER) using the other strand as a template. As both strands are compromised in ICLs, other enzymes are involved in their repair. Two major pathways of ICL repair exist; one is replication dependent and mainly involves homologous recombination, the second is replication independent and involves NER (Ho and Schärer, 2010). At the start of both of these pathways, translesion (TLS) polymerases are needed to bypass ICLs and restore one of the two DNA strands. Translesion synthesis is a mechanism used by cells to prevent common DNA damage from stalling replication forks and rising apoptosis levels. The most important TLS polymerases are Pol ζ (Polymerase zeta) and REV1 (Reversionless 1). Studies have shown that disruption or suppression of expression of both *REV3L*, the gene encoding the catalytic subunit of Pol ζ, or *REV1* modifies sensitivity to cisplatin (Lin et al., 2006; Doles et al., 2010). Goricar et al. (2014) recently determined in patients with malignant mesothelioma that the mutant allele in *REV1* rs3087403 and *REV1* TGT haplotype associated with increased risk for leukopenia and neutropenia. *REV3L* rs465646, rs462779, and *REV3L* CCGG haplotype associated with longer overall survival (Goricar et al., 2014).

### DNA repair enzymes

DNA damage repair mechanisms are as follows: direct repair of alkyl adducts; repair of base damage and single strand breaks by base excision repair; repair of double strand breaks by homologous recombination or by non-homologous end joining; repair of bulky DNA adducts by NER; and repair of mismatches and insertion/deletion loops by DNA mismatch repair (Camps et al., 2007). The NER pathway is one of the major DNA repair systems involved in the removal of platinum adducts. This pathway involves many proteins in lesion recognition, excision, DNA synthesis and ligation. Excision repair cross-complementary 1 (ERCC1) is a key protein involved in the process of NER and ERCC1-xeroderma pigmentosum (ERCC1-XPF) catalyzes incision on the incision 50 side to the site of DNA damage (Parker et al., 1991; Bessho, 1995). In addition to ERCC1, xeroderma pigmentosum complementary group D (XPD) encodes a helicase that participates in both NER and basal transcription as part of the transcription factor, IIH. Mutations destroying the enzymatic function of XPD protein are manifested clinically in combinations of three severe syndromes, including xeroderma pigmentosum, XP combined with Cockayne Syndrome and trichothiodystrophy (Lehmann, 2001; Clarkson and Wood, 2005). ERCC1 and ERCC2 (XPD) have pivotal roles in the NER pathway, this has been evidenced in studies where lower levels of intratumoral ERCC1 mRNA are significantly correlated with improved survival due to enhanced tumor cell sensitivity to cisplatin (Shirota et al., 2001). mRNA levels as well as the overexpression of ERCC1 and other enzymes have been implicated in the development of clinical resistance to platinum (Kirschner and Melton, 2010; Cheng et al., 2012).

Among these genes, the most studied is *ERCC1* gene, mostly focused on the therapy of non-small cell lung cancer (NSCLC) and esophageal cancer. Polymorphisms in *ERCC1* include mainly rs3212986 and rs11615. The polymorphism rs3212986 is located in the 3′ untranslated region and therefore may affect mRNA stability resulting in a decreased expression levels (Chen et al., 2000). In relation to rs3212986, the C allele leads to a change that results in an increase in overall survival (Zhou et al., 2004; Krivak et al., 2008; Takenaka et al., 2010), progression free survival (Krivak et al., 2008; Kim et al., 2009; Erčulj et al., 2012; Chen et al., 2013), treatment response (Li et al., 2010) and prognosis (Takenaka et al., 2010; Okuda et al., 2011). However, opposite associations have been reported in other studies related to reduced responses with the C allele (Bradbury et al., 2009; Kalikaki et al., 2009; Park et al., 2011; Wang et al., 2011), as well as increased toxicity (Khrunin et al., 2010; Tzvetkov et al., 2011; Erčulj et al., 2012). Wang et al. (2011) and Bradbury et al. (2009) showed that in esophageal cancer, patients with A/A or A/C genotype had improved outcomes compared with patients carrying wild-type genotypes. In addition, Park et al. (2011) have found similar results in metastatic cancer patients. On the contrary, opposite results have been found in NSCLC and ovarian cancer where the C allele relates to improved survival and treatment response. The variability in outcomes amongst these studies could be due to tumor characteristics (tissue-specific or organ-specific). The polymorphism C→T at codon 118 located on exon 4 of *ERCC1* gene (rs11615) is expected to have the same effect. This polymorphism is associated with clinical response to platinum-based chemotherapy in NSCLC. The C allele is also related to an increase in overall survival (Isla et al., 2004; Ryu et al., 2004; Cheng et al., 2012; Joerger et al., 2012), progression free survival (Ryu et al., 2004; Cheng et al., 2012; Joerger et al., 2012), improved treatment response (Kalikaki et al., 2009) and prognosis (Okuda et al., 2011). Nevertheless, others authors detect opposite associations in larger-population studies, including amongst Chinese patients (Li et al., 2010; Ren et al., 2012): this should be considered in future research. Nephrotoxicity has been related to the C allele in rs3212986 *ERCC1* (Tzvetkov et al., 2011), T allele in rs11615 *ERCC1* (Tzvetkov et al., 2011) and C/T genotype in rs3212986 *ERCC1* (Khrunin et al., 2010), independent of cancer type.

Another widely studied gene is *ERCC2* (*XPD*). The presence of a variation in *ERCC2* gene (rs13181 and rs1799793) reduces repair capacity, and results in greater efficacy of cisplatin treatment due to increased DNA damage and an enhanced cytotoxic effect. rs1799793 generates a positive effect in overall survival and progression free survival (Gurubhagavatula et al., 2004; Bradbury et al., 2009; Biason et al., 2012). Erčulj et al. (2012) found that G/G genotype is related to an increase in various types of toxicity (Erčulj et al., 2012) while nephrotoxicity has been shown by Joerger et al. (2012) (Joerger et al., 2012). The A allele in the mutation rs13181 increases overall survival (Park et al., 2001; Quintela-Fandino et al., 2006; Caronia et al., 2009; Chew et al., 2009). However, other authors have found the C allele related to increased overall survival (Bradbury et al., 2009) in esophageal cancer and progression free survival in pancreatic cancer (Avan et al., 2013). These discrepancies suggest that associations with C allele are not fully clear in these types of cancers, and that patients factors, treatment modalities and ethnic population could influence the outcome. Nonetheless, the majority of the results support an association between both rs1799793 and rs13181 and clinical outcomes in patients with NSCLC, osteosarcoma, breast cancer, ovarian cancer, and colorectal cancer. These significant associations in *ERCC2* polymorphisms and clinical outcomes have included studies with a larger number of patients and differing patient populations.

Other studies found associations between *ERCC5* mutations (rs1047768 and rs751402), PFS (progression free survival) (Sun et al., 2013) and OS (overall survival) (He et al., 2013). These studies have indicated that *ERCC5* polymorphisms are involved in the efficacy of cisplatin neoadjuvant chemotherapy. Also, ototoxicity has related to rs2228001 mutation in the Xeroderma Pigmentosum Complementation group C (*XPC*) gene (Caronia et al., 2009). More information is needed about these associations to reach more powerful conclusions, including a greater number of patients and amongst different ethnic populations.

Additional DNA repair genes have also shown variability, including X-ray repair cross-complementing group 1 (*XRCC1*). This protein is involved in base excision repair. Among the mutations, we highlight rs25487 and rs1799782 mutations. In relation to rs25487, the mutant G variant has been associated with decreased treatment response (Gurubhagavatula et al., 2004; Giachino et al., 2007; Pacetti et al., 2009; Khrunin et al., 2010; Joerger et al., 2012; Ke et al., 2012; Miao et al., 2012), although opposite results exist (Quintela-Fandino et al., 2006; Sakano et al., 2006). Other evidence indicates associations between the G allele and neutropenia (Khrunin et al., 2010). T allele in rs1799782 mutation is related with an increase (Miao et al., 2012; Li and Li, 2013) and decrease in overall survival (Li et al., 2006; Shim et al., 2010). Li and Li (2013) and Miao et al. (2012) have performed studies in ovarian cancer with a large number of patients. Further data are required to confirm this association. Another finding is the relation between treatment response and the T allele (Wang et al., 2004; Yuan et al., 2006; Kim et al., 2009; Ke et al., 2012). This discrepancy may be due to cancer type or combined therapies. DNA repair enzymes might decrease the synergistic effects of combination of cisplatin and radiation and information from population should be added in future association specifics to subgroups (Li and Li, 2013). In addition, some studies have used cisplatin in combination with paclitaxel, gemcitabine, cyclophosphamide or 5-FU, depending on cancer type. Others factors that might affect variability in different populations are the stage of disease, patient status and period of follow-up in survival analysis.

With respect to X-ray repair cross complementing protein 3 (XRCC3), a protein involved in DNA double-strand breaks, the rs861539 mutation is the only one that relates to treatment outcome. Increased overall survival was associated with the T allele (De las Peñas et al., 2006; Chen et al., 2012) as was progression free survival (Font et al., 2008). However, Ren et al. (2012) have shown inverse results (Ren et al., 2012) including a large number of patient (*n* = 340) with NSCLC. More data are necessary to confirm these opposing results.

In summary, studies of association between genetic variants in the DNA repair system and clinical results show that these variants can be potential biomarkers for outcomes in the cisplatin-based therapies (Table 1). Despite race and treatment regimen, associations testing the polymorphism in *ERCC1* appear to follow a consistent direction. rs3212986 and rs11615 polymorphisms should be considered in a future genetic panel because results were obtained in several researches with different treatment and demographic characteristics. Additional research should be performed in order to replicate results found with polymorphisms in *ERCC2, XRCC1*, and *XRCC3*. In additional studies, the later polymorphism should be used to evaluate clinical outcomes (overall survival and disease progression) considering different subgroups of patient. In relation to specific toxicities, associations with nephrotoxicity have been described and characterized, but likewise require confirmation.

**Table 1:** **Summary of association studies between genetic polymorphisms and outcomes in the cisplatin-based chemotherapy**.

**Gene**	**Mutation**	**Cancer**	**References**	**Number of subjects**	**Results**
*ERCC1*	Gln504Lys	NSCL	Kalikaki et al., 2009	119	C/C ↓ OS
	rs3212986		Nigro et al., 2010	7	Related with survival
	NC_000019.10:g.45409478C>A		Okuda et al., 2011	90	C/C ↑ Prognosis
	NG_015839.2:g.74351G>T		Takenaka et al., 2010	122	C/C ↑ DFS and OS
			Zhou et al., 2004	128	C/C ↑ OS
			Li et al., 2010	115	C→ A ↓ Response
		Advanced esophageal cancer	Wang et al., 2011	241	C/C ↓ Remission rate and PFS
			Bradbury et al., 2009	262	Related with OS
			Rumiato et al., 2013	143	Related outcomes
		Nasopharyngeal cancer	Chen et al., 2013	101	C/C ↓ Risk of progression
			Liu et al., 2013	104	C/C ↓ PFS
		Epithelial ovarian cancer	Kim et al., 2009	118	C/A or A/A ↓ PFS and OS
			Krivak et al., 2008	233	C/C ↑ PFS and OS
			Khrunin et al., 2010	104	C/A ↑ Risk of nephrotoxicity
		Malignant mesothelioma	Erčulj et al., 2012	133	C/C ↑ PFS, Risk of toxicity
		Cisplatin-treated cancer	Tzvetkov et al., 2011	79	C allele ↓ eGFR (Nephrotoxicity)
		Metastatic gastric cancer	Park et al., 2011	108	C/C ↓ Response rate and Time to progression
	Asn118Asn	NSCLC	Cheng et al., 2012	142	C/C ↑ Response rate, PFS and OS
	rs11615		Joerger et al., 2012	137	C/C ↑ Response rate, PFS and OS
	NC_000019.10:g.45420395A>G		Okuda et al., 2011	90	C/C ↑ Prognosis
	NG_015839.2:g.63434T>C		Ryu et al., 2004	109	C/C ↑ Survival
			Isla et al., 2004	62	C allele ↑ Survival
			Li et al., 2010	115	C→ T ↑ Response
			Su et al., 2007	230	T allele ↑ Response
			Ren et al., 2012	340	C/C ↓ survival
			Kalikaki et al., 2009	119	C/C, C/T ↑ Response
		Advanced esophageal cancer	Warnecke-eberz et al., 2009	52	T/T ↑ Response
		Epithelial ovarian cancer	Smith et al., 2007	103	C/C ↑ Progression and death
		Osteosarcoma	Hao et al., 2012	267	T/T ↑ Event free survival
		Esophageal adenocarcinoma	Metzger et al., 2012	217	C/C ↓ Response
		Melanoma	Liu et al., 2005	90	C/C ↓ Response
		Pancreatic cancer	Kamikozuru et al., 2008	67	T allele ↑ PFS and OS
		Cisplatin-treated cancer	Tzvetkov et al., 2011	79	T allele ↓ eGFR (Nephrotoxicity)
	Haplotype	Advanced gastric cancer	Goekkurt et al., 2009	156	T allele/C allele ↑ grade 3-4 neutropenia
	rs3212986/rs11615				
*ERCC2 (XPD)*	Asp312Asn	Esophageal cancer	Bradbury et al., 2009	262	Related with OS
	rs1799793				
	NC_000019.10:g.45364001C>T				
	NG_007067.2:g.11587G>A				
		Malignant mesothelioma	Erčulj et al., 2012	133	G/G ↑ Risk of toxicity
		Ovarian cancer	Khrunin et al., 2010	104	G/G ↑ Severe neutropenia
		NSCLC	Gurubhagavatula et al., 2004	103	A allele ↓ OS
			Joerger et al., 2012	137	A allele related with OS
		Squamous cell carcinoma of the head and neck	Quintela-Fandino et al., 2006	103	A allele ↑ OS
		Osteosarcoma	Biason et al., 2012	130	G/A or A/A ↑ Response
	Lys751Gln	Esophageal cancer	Bradbury et al., 2009	262	Related with OS
	rs13181				
	NC_000019.10:g.45351661T>G				
	NG_007067.2:g.23927A>C				
		Pancreatic cancer	Avan et al., 2013	122	Related with risk of death
		Colorectal cancer	Park et al., 2001	73	A/A ↑ response
		NSCLC	Chen et al., 2012	355	A/A ↑ OS
			Ren et al., 2012	340	A/A ↑ OS
			Ludovini et al., 2011	192	C/C ↑ PFS
		Osteosarcoma	Caronia et al., 2009	91	Allele G ↓ Response
			Hao et al., 2012	267	A/A ↑ Event free survival
		Squamous cell carcinoma of the head and neck	Quintela-Fandino et al., 2006	103	C allele ↑ OS
		Breast cancer	Chew et al., 2009	55	Related with clinical outcomes
	Haplotype (rs1799793 /rs13181)	Advanced gastric cancer	Goekkurt et al., 2009	156	Related with nephrotoxicity
	rs50872	NSCLC	Kim et al., 2012	129	A/A ↓ OS
	NC_000019.10:g.45359191A>G				
	NG_007067.2:g.16397T>C				
	Asp711Asp	NSCLC	Li et al., 2013	496	C/T + T/T ↓ Response
	rs1052555				
	NC_000019.10:g.45352266G>A				
	NG_007067.2:g.23322C>T				
*XPC*	Lys939Gln	Osteosarcoma	Caronia et al., 2009	91	C/C association with ototoxicity
	rs2228001				
	NC_000003.12:g.14145949G>T				
	NG_011763.1:g.37724C>A				
*ERCC5*	rs1047768	Osteosarcoma	Sun et al., 2013	182	T/T ↑ PFS and OS
	NC_000013.11:g.102852167T>C				
	NG_007146.1:g.11344T>C				
	rs751402	NSCLC	He et al., 2013	228	A/A ↓ Response
	NC_000013.11:g.102845848A>G				
	NG_007146.1:g.5025A>G				
*XRCC1*	Gln399Arg	Ovarian cancer	Chung et al., 2006	36	A allele ↓ Response
	rs25487		Khrunin et al., 2010	104	G/G ↓ Severe neutropenia
	NC_000019.10:g.43551574T>C				
	NG_033799.1:g.29005A>G	NSCLC	Gurubhagavatula et al., 2004	103	A allele ↓ OS
			Joerger et al., 2012	137	G allele related with OS
			Wang et al., 2004	105	Gallele ↑ Response rate
			Giachino et al., 2007	203	A/A ↑ Median Survival Time
			Ke et al., 2012	460	A/A ↑ Survival
			Lee et al., 2013	382	A allele ↓ Response
		Advanced gastric cancer	Goekkurt et al., 2009	156	Related with OS
			Ji et al., 2013	59	A/A ↑ OS
		Nasopharyngeal cancer	Zhai et al., 2013	60	A/A related with remission
		Ovarian cancer	Li and Li, 2013	335	A/A ↑ Risk of death
			Miao et al., 2012	195	A/A ↑ Risk of death
		Ovarian cancer	Khrunin et al., 2010	104	A/A Severe neutropenia
		Musculoskeletal cancer	Sakano et al., 2006	78	G/A + A/A ↑ OS
		Squamous cell carcinoma of the head and neck	Quintela-Fandino et al., 2006	103	A allele ↑ OS
		Biliary tract carcinoma	Pacetti et al., 2009	33	G/G ↓ OS
	Arg194Trp rs1799782	Pancreatic cancer	Li et al., 2006	92	T allele ↓ Survival
	NC_000019.10:g.43553422G>A				
	NG_033799.1:g.27157C>T				
		NSCLC	Sun et al., 2009	82	C/T ↑ Response
			Wang et al., 2004	105	C/T or T/T ↑ Response
			Hong et al., 2009	164	C/T + T/T ↑ Response
			Ke et al., 2012	460	T/T ↓ Risk of death
		Ovarian cancer	Li and Li, 2013	335	T/T ↑ OS
			Miao et al., 2012	195	T/T ↑ Risk of death
		Cervical cancer	Kim et al., 2008	66	C/C ↓ Response
		Gastric cancer	Shim et al., 2010	200	C/T ↓ OS
	Haplotype	NSCLC	Hong et al., 2009	164	A - T haplotype ↑ Response
	(rs25487/rs1799782)				
*XRCC3*	Thr241Met rs861539	NSCLC	De las Peñas et al., 2006	135	T/T ↑ OS
	NC_000014.9:g.103699416G>A		Ren et al., 2012	340	C/C ↑ OS
	NG_011516.1:g.21071C>T NG_012307.1:g.75229G>A	Breast cancer	Chew et al., 2009	136	C/C ↑ Response rate and PFS
		Advanced esophageal cancer	Font et al., 2008	28	T/T ↑ TTP
		Adenocarcinoma of esophageal and stomach	Ott et al., 2011	258	T allele ↑ OS

*Polymorphisms on DNA repair*.

*OS, Overall survival; PFS, Progression free survival; TTP, Time to progression; EFS, Event free survival; DFS, Disease free survival; G-CSF, Granulocyte-Colony Stimulating Factor; eGFR, Glomerular filtration rate*.

## Pharmacokinetic mechanisms

Evidence indicates that reduced drug accumulation is a significant mechanism of cisplatin resistance (Kelland, 1993). The cause may be an inhibition in drug uptake, an increase in drug efflux, or both. Studies concerning the mechanisms of cisplatin uptake into the cell have focused on both passive diffusion (Hromas et al., 1987; Binks and Dobrota, 1990; Mann et al., 1991) and copper transporters (Katano et al., 2002; Ohashi et al., 2003; Safaei et al., 2004).

Recent studies have demonstrated that mutation or deletion of the *CTR1* gene results in increased cisplatin resistance and reduction of platinum levels (Ishida et al., 2002). Copper-transporting P-type adenosine triphosphate (ATP7B) is associated with cisplatin resistance *in vitro* (Komatsu et al., 2000), and in various cancers (Nakayama et al., 2002, 2004; Ohbu et al., 2003). ATP-binding cassette sub-family C2 (ABCC2), another transporter protein, also has a role in cisplatin resistance, probably promoting drug efflux (Koike et al., 1997; Kool et al., 1997; Cui et al., 1999). ABCC3 is a member of the multidrug resistance protein (MRP) family. Caronia et al. (2011) found that rs4148416 was associated with low survival. In addition, the ABCB1 gene that is well-known and encodes P-glycoprotein, contains three polymorphisms (rs2032582, rs1045642, and rs1128503) that have been studied individually and as a haplotype, however, the results have been inconsistent (Caronia et al., 2011).

### Detoxification

Cisplatin is inactivated by conjugation with glutathione through the GSTs. This phase II enzyme catalyzes the conjugation of reactive metabolites with negatively charged hydrophilic molecules for disposal in excretion processes. Genetic variations in GSTs have been implicated in cellular resistance to cancer chemotherapy and in outcomes of cisplatin-based treatments. When GSTs enzymes with reduced activity are present, the available concentration in the drug in tumor tissue increases. In these patients therapy might be more effective, but might also be severely toxic (Strange et al., 2000; Siddik, 2003; Quiñones et al., 2006). Several studies have shown significant association between polymorphic *GSTs* genes and cisplatin treatment response suggesting these polymorphisms as potential biomarkers (Table 2).

**Table 2:** **Summary of association studies between genetic polymorphisms on Glutathione-S-Transferases and outcomes in the cisplatin-based chemotherapy**.

**Gene**	**Mutation**	**Cancer**	**References**	**Number of subjects**	**Results**
*GSTP1*	Ile105Val	Testicular cancer	Oldenburg et al., 2007b	173	G/G ↓ Ototoxicity
	rs1695	Testicular cancer	Oldenburg et al., 2007a	238	G/G ↓ neurotoxicity
	NC_000011.10:g.67585218A>G	Ovarian cancer	Khrunin et al., 2010	104	A/A ↑ OS and PFS
	NG_012075.1:g.6624A>G	Urothelial cancer	Yokomizo et al., 2007	179	G allele ↑ myelosuppression
		Epithelial ovarian cancer	Kim et al., 2009	118	A/A ↑ Risk for grade 3 or 4 Hematological Toxicity
		Advanced gastric cancer	Ji et al., 2013	59	G/G ↑ Survival
			Goekkurt et al., 2009	156	A/A ↑ Grade 3-4 neutropenia and neurotoxicity
			Ruzzo et al., 2006	175	A/A ↓ Survival
		Osteosarcoma	Windsor et al., 2012	60	G Allele ↑ Myelosuppression
			Yang et al., 2012	187	G Allele ↑ Rates of response
		NSCLC	Joerger et al., 2012	137	G/G ↑ Risk of polyneuropathy
			Sun et al., 2010	113	G Allele ↑ Response
		Medulloblastoma	Rednam et al., 2013	106	G Allele ↑ ototoxicity
		Gastric cancer	Goekkurt et al., 2006	52	G/G ↑ survival
*GSTA1*	rs3957357	Ovarian cancer	Khrunin et al., 2010	104	T/T ↑ Survival vs. C/C
	NC_000006.12:g.52803889A>G				
	NM_145740.3:c.-135T>C				
*GSTT1*	Null	Epithelial ovarian cancer	Kim et al., 2009	118	Non-null ↓ OS, PFS
		Advanced gastric cancer	Goekkurt et al., 2009	156	Non-null ↑ OS and PFS
		Platinum chemotherapy	Jurajda et al., 2012	55	Null allele ↑ onset of ototoxicity
		Pediatric solid tumor	Choeyprasert et al., 2013	68	Non-null related with ototoxicity
*GSTM1*	Null	Ovarian cancer	Beeghly et al., 2006	215	Null allele ↑ OS
			Khrunin et al., 2010	104	Null allele ↓ Thrombocytopenia, anemia and neuropathy
		Neck and head cancer	Dhawan et al., 2013	23	Null allele ↑ Toxicity
		Breast cancer	Petros et al., 2005	85	Null allele ↑ OS
		Testicular cancer	Oldenburg et al., 2007b	173	Non-null ↑ ototoxicity
			Oldenburg et al., 2007a	238	Non-null ↑ ototoxicity
					Null allele ↓ ototoxicity
		Advanced ovarian cancer	Medeiros et al., 2003	24	Null allele ↑ PFS and OS
			Ott et al., 2008	139	Null allele ↑ OS
*GSTM3*	rs1799735	Cisplatin-based chemotherapy	Peters et al., 2000	19	Deletion in intron 6 ↓ ototoxicity
	NC_000001.10:g.110280254delC, NC_000001.10:g.110280254delCinsCCT				
		Cisplatin-based chemotherapy	Khrunin et al., 2010	104	AGG/AGG ↓ Thrombocytopenia, anemia and neuropathy

*OS, Overall survival; PFS, Progression free survival; TTP, Time to progression; EFS, Event free survival; G-CSF, Granulocyte-Colony Stimulating Factor*.

In the GSTs superfamily there are eight cytosolic classes (Alpha, kappa, mu, omega, pi, sigma, theta, and zeta) (Katoh et al., 2008; Luo et al., 2011). *GSTP1, GSTM1*, and *GSTT1* genes, have been the most widely studied in relation to the functional polymorphisms. *GSTP1* is widely expressed in normal human epithelial tissues. A single nucleotide substitution (A→G) at position 313 (rs1695) of the *GSTP1* gene, results in replacement of isoleucine with valine at codon 105 of the enzyme, substantially diminishes GSTP1 enzyme activity. On the contrary, *GSTM1* and *GSTP1* genetically delected (homozygous null allele) will lead to an absence of enzymatic activity (Stoehlmacher et al., 2002).

The *GSTP1* gene has been the most studied in a wide number of cancers with controversial results related to cisplatin-based therapy. Some investigations have shown that patients with G/G genotype present less toxicity (Oldenburg et al., 2007a,b; Goekkurt et al., 2009; Kim et al., 2009) with more survival (Goekkurt et al., 2006; Ruzzo et al., 2006; Ji et al., 2013) and better therapy response (Sun et al., 2010; Yang et al., 2012). On the other hand, the G allele has been associated with a risk of myelosuppression, polyneuropathy, and toxicity (Yokomizo et al., 2007; Joerger et al., 2012; Windsor et al., 2012; Rednam et al., 2013). In ovarian cancer, the A allele is related to better PFS and OS (Khrunin et al., 2010). *GSTP1* A/A genotype has been found to predict suboptimal response to flurouracil/cisplatin chemotherapy and poor survival in patients with advanced gastric cancer (Ruzzo et al., 2006). The influence of rs1695 *GSTP1* on toxicity to taxane-and platinum-based chemotherapy is in debate (Kim et al., 2009).

Polymorphism of *GSTM1* and *GSTT1* genes is associated with cisplatin-based treatments. *GSTM1* null has been specifically related to an increase of OS and PFS (Medeiros et al., 2003; Petros et al., 2005; Beeghly et al., 2006; Ott et al., 2008). Concerning toxicity, it has been associated with a decrease in toxicity (Oldenburg et al., 2007a,b; Khrunin et al., 2010), although Dhawan et al. (2013) showed the opposite but with a small sample (*n* = 23) (Dhawan et al., 2013). On the *GSTT1* gene, the non-null allele relates to an increase in overall survival and progression free survival (Goekkurt et al., 2009), however, Kim et al. (2009) showed the opposite but this contradiction apparently is caused by different definitions of patient response. Moreover, the null allele has also associated with an increase in ototoxicity (Jurajda et al., 2012; Choeyprasert et al., 2013). Finally, additional studies examining the *GSTA1* gene showed the T/T genotype (rs3957357) associates with an increase of overall survival (Khrunin et al., 2010). Regarding to *GSTM3* gene, the AGG/AGG haplotype (rs1799735) is related to less thrombocytopenia, anemia and neuropathy (Khrunin et al., 2010). Nevertheless, more evidence is needed in order to determine a clear role of *GSTA1* and *GSTM3* genes on cisplatin-based therapy.

Polymorphisms in the *GSTP1* gene have shown controversial results among different types of cancer. Some studies found the polymorphic allele related to less toxicity, better therapy response and more survival but others found the opposite regarding to toxicity (Rednam et al., 2013). The results obtained by several authors demonstrate that the *GSTM1* null allele is consistently related to overall survival in different types of cancer. Concerning toxicity, few investigation have found associations, therefore the role of this polymorphism on toxicity is not clear. On the other hand, the *GSTT1* null allele associates with toxicity in patients carrying this polymorphism. Regarding OS and PFS it appears that null allele is related to decreased OS and PFS, although one author showed the opposite (Ruzzo et al., 2006; Goekkurt et al., 2009). This contradiction apparently is caused by different definitions of patient response.

Together, the evidence appears to indicate a strong association between *GSTs* polymorphisms and clinical response (overall survival and disease progression). However, the effects on toxicity do not appear to have a clear and dominant trend, and may be related to differing treatment modalities in each of the studies. Despite this, with the data presented we can conclude that the *GSTP1* polymorphic allele and the *GSTM1* and *GSTT1* null alleles appear to result in enhanced overall survival and progression free survival, particularly in gastric cancer where the data have been more consistent. Lack of activity in GSTs enzymes appear to lead to a better treatment response.

## Conclusion

Personalized therapy promises improved outcomes to treatment with respect to efficacy and toxicity of treatment. Ideally, sub-groups of patients that would require adjustment to therapy based on genetic information could be detected prior to commencing treatment, and therapy accordingly optimized. Pharmacogenetics, the study of the role of inheritance in individual variation in drug response, can address cisplatin cellular resistance, providing tools to achieve the modification of current treatments in different types of cancer, including lung, gastric, ovarian, testicular and, esophageal cancers (Weinshilboum, 2003).

Variable responses to different treatments, including cisplatin, have been seen from different points of view. When looking into the genetic variability in processes where cisplatin is involved, including pharmacokinetics and pharmacodynamics, efforts have delivered evidence regarding DNA repair systems and metabolization systems. Within the variability in DNA repair processes, key genes involved include *ERCC1, ERCC2* (*XPD*), *ERCC5, XRCC1, XRCC3*, and *XPC* genes. Studies examining the genetic variability of cisplatin metabolism have shown that the main genes involved are *GSTP1, GSTM3, GSTM1*, and *GSTT1*. Currently there appears to be a group of genes that would influence variability in response and toxicity in cisplatin-based therapies which we present here in this up-dated review.

Diverse results have been found among the polymorphisms analyzed in both DNA repair enzymes and detoxification enzymes. These contradictions and variations are primarily due to the heterogeneity amongst studies (patient population, treatment and number of subjects). Another possibility is with the inclusion of a large number of candidate genes, there is always a risk of false positive associations. For example, recent studies showed a relationship between rs12201199 in thiopurine S-methyltransferase gene (*TPMT*) and rs9332377 in the catechol-O-methyltransferase gene (*COMPT*) with cisplatin-induced hearing loss in children (Ross et al., 2009). Our opinion is that future studies in this line should include the genes we have highlighted, and that a collaborative effort is required to improve the quality and strength of evidence in order to achieve a validated panel of polymorphisms that guides therapeutic decisions.

Finally, prospective clinical studies employing polymorphism panels in these treatment procedures are required to determine whether adjustment of therapy based on genetic information can influence outcomes in these scenarios.

## Author contributions

Ángela Roco: Review of intellectual content and Final approval, Juan Cayún: Substantial contributions, Stephania Contreras: Substantial contributions, Jana Stojanova: Substantial contributions, Luis Quiñones: Review of intellectual content and Final approval.

### Conflict of interest statement

The authors declare that the research was conducted in the absence of any commercial or financial relationships that could be construed as a potential conflict of interest.
